# Molecular Analysis of *Colletotrichum* Species in the Carposphere and Phyllosphere of Olive

**DOI:** 10.1371/journal.pone.0114031

**Published:** 2014-12-11

**Authors:** Saveria Mosca, Maria G. Li Destri Nicosia, Santa O. Cacciola, Leonardo Schena

**Affiliations:** 1 Dipartimento di Agraria, Università Mediterranea, Reggio Calabria, Italy; 2 Dipartimento di Gestione dei Sistemi Agroalimentari e Ambientali, Università degli Studi, Catania, Italy; University Hospital of the Albert-Ludwigs-University Freiburg, Germany

## Abstract

A metagenomic approach based on the use of genus specific primers was developed and utilized to characterize *Colletotrichum* species associated with the olive phyllosphere and carposphere. Selected markers enabled the specific amplification of almost the entire ITS1-5.8S-ITS2 region of the rDNA and its use as barcode gene. The analysis of different olive samples (green and senescent leaves, floral residues, symptomatic and asymptomatic fruits, and litter leaves and mummies) in three different phenological phases (June, October and December) enabled the detection of 12 genotypes associated with 4 phylotypes identified as *C. godetiae, C. acutatum s.s., C. gloeosporioides s.s*. and *C. kahawae*. Another three genotypes were not identified at the level of species but were associated with the species complexes of *C. acutatum*, *C. gloeosporioides* and *C. boninense sensu lato*. *Colletotrichum godetiae* and *C. acutatum s.s.* were by far the most abundant while *C. gloeosporioides s.s.* was detected in a limited number of samples whereas ther phylotypes were rarely found. The high incidence of *C. acutatum s.s.* represents a novelty for Italy and more generally for the Mediterranean basin since it had been previously reported only in Portugal. As regards to the phenological phase, *Colletotrichum* species were found in a few samples in June and were diffused on all assessed samples in December. According to data new infections on olive tissues mainly occur in the late fall. Furthermore, *Colletotrichum* species seem to have a saprophytic behavior on floral olive residues. The method developed in the present study proved to be valuable and its future application may contribute to the study of cycle and aetiology of diseases caused by *Colletotrichum* species in many different pathosystems.

## Introduction

The fungal genus *Colletotrichum* comprises many plant pathogens of relevant economic significance as they cause major diseases on a wide variety of plant species. It is primarily diffused in tropical and subtropical areas, but crops in temperate areas can also be severely affected [1]. *Colletotrichum* species are commonly associated with anthracnose symptoms and mainly involve fruits and aerial plant parts. Serious diseases caused by *Colletotrichum* species include red rot of sugar cane, coffee berry disease, crown rot of strawberry and banana, and brown blotch of cowpea. Besides, olive anthracnose, is the most damaging disease of olive fruit worldwide and severely affects both fruit yield and quality of oil which is characterized by off-flavor, reddish color, high acidity, and a considerable reduction of β-sitosterol, polyphenols and α-tocopherol [2], [3]. In moist conditions, infected olives show a soft to dark brown rot with an abundant production of orange conidia while, in dry conditions, the drupes mummify. The disease is caused by different species of the genus *Colletotrichum* primarily belonging to two complexes of species showing high phenotypic and genotypic diversity, *C. acutatum sensu lato* (*s.l.*) and *C. gloeosporioides s.l.* [2], [4]–[6]. Recently, *C. karstii*, a species belonging to the *C. boninense* complex has also been associated with olive anthracnose, but it does not seem to constitute a serious threat for olive production [7], [8].

A number of studies conducted on *C. acutatum s.l.* isolates associated with olive anthracnose have revealed that six different species [*C. simmondsii, C. fioriniae, C. godetiae* (syn. *C. clavatum*), *C. acutatum sensu stricto* (s.s.), *C. nymphaeae* and *C. rhombiforme*] formerly identified as species groups are primarily involved in the disease [3]–[5], [7], [9]–[11]. Available data indicate *C. godetiae* as the prevalent species in Greece, Montenegro, some restricted areas of Portugal and Italy with two vegetative compatibility groups (VCGs) that have a distinct geographic distribution [11]. On the contrary, *C. nymphaeae* is the predominant species in Portugal (80% of the isolates), followed by *C. godetiae* (12% of the isolates) and *C. acutatum s.s.* (3–4% of the isolates). However, in another region of Portugal, four different species (*C. nymphaeae, C. fioriniae, C. godetiae* and *C. acutatum s.s*.) were found and no single sub-population was dominant [12]. *C. acutatum s.s*., which has not been isolated from olive in Italy so far, is the prevalent species in olive-growing areas in Australia and South Africa while *C. godetiae* is the dominant pathogen in Andalusia, Spain [5], [13]–[16].

*Colletotrichum gloeosporioides* has rarely been associated with epidemic explosions of the disease and is commonly considered as a less aggressive pathogen [11], [12]. However, symptoms on infected olives were also induced by some virulent isolates of this species. Schena and co-workers [8] found that seven different species, recently separated within the *C. gloeosporioides s.l.* complex (*C. aenigma, C. gloeosporioides s.s., C. kahawae, C. queenslandicum, C. siamense* and *C. theobromicola*) can cause olive anthracnose, and one of these (*C. theobromicola*) seems to represent a serious threat for olive productions in Australia [8]. It was speculated that contrasting reports on the virulence of *C. gloeosporioides* isolates could be partially due to the improper former identification of isolates, mainly based on morphological and cultural criteria.

Currently available data regarding aetiology, biology and epidemiology of *Colletotrichum* species associated with olive anthracnose are based on fungal isolations and subsequent identifications using morphological features and molecular markers [4], [8], [11]. These methods are time-consuming and costly. Furthermore, sampling bias introduced by the isolation phase may be a limitation and cause a serious underestimation of the pathogen diversity within host tissues since fast-growing fungal species can frequently conceal the presence of *Colletotrichum*. Since effective selective media are not available for the genus *Colletotrichum*, isolations can be quite easy in the case of fruit infections because external contaminating microorganisms can be physically avoided. On the other hand, isolations are much more complicated in the case of thin organs like leaves, flowers and fertilized fruits as well as in the case of decayed organs such as litter leaves and fruits.

The characterization of environmental fungal communities by means of metagenomic approaches represents a powerful emerging strategy since it facilitates the detection and analysis of all fungi (cultivable and uncultivable) and enables a more realistic assessment of the genetic diversity in microbial communities [17]. Numerous studies based on the PCR amplification of single target regions to investigate “*in situ*” microbial communities have unexpectedly revealed diverse fungal populations and highlighted the existence of previously unknown lineages in a number of different ecosystems [18]. The internal transcribed spacer (ITS) region of the ribosomal DNA (rDNA) has been selected as the universal barcode for fungi because it can be easily amplified and sequenced and, compared to alternative genes, it has the highest probability of successful identification for a broader range of fungi. The ITS region includes the variable ITS1, the conserved 5.8S and the variable ITS2 and is situated between the small (SSU) and the large (LSU) subunits of the rDNA. In recent years, a number of studies have focused on the design of appropriate universal primers for the *in situ* amplification of one or both ITS1 and ITS2 regions from all fungal species for metagenomic analyses [19], [20]. The use of these universal primers represent a valuable tool for ecological studies since it enables the characterization of the whole fungal genetic diversity in a given environment, but could also represent a limit in the case of more specific studies such as those focusing on specific fungal genera. Indeed, fungal plant pathogens may represent a minority if compared to the complete fungal diversity [21], [22]. In this context, the use of more selective primers can greatly facilitate and improve analyses as recently demonstrated for the genera *Phytophthora* and *Trichoderma* [23], [24].

The aim of the present study was the development and application of a molecular method based on genus specific primers for the analysis of *Colletotrichum* diversity in the olive phyllosphere and carposphere.

## Materials and Methods

### Ethics statement

No specific permits were required for the described field studies. This work did not involve endangered or protected species.

### DNA extraction

DNA extractions from pure fungal cultures were made according to the protocol described by Schena et al. [25] except for the use of lyophilized mycelium (2 mg) instead of fresh mycelium. Extracted DNA was suspended in RNase free water and stored at −20°C. For routine analyses DNA was diluted to 50 ng/µl and maintained at 4°C.

Field-collected olive samples were quickly returned to the laboratory in plastic containers and stored in a cold room at 4°C for no longer than 24 h prior to lyophilization. Before lyophilization leaves were cut into small pieces of approximately 5–10 mm while drupes collected in October and December were separated from the stone, which was discarded. Floral residues and fertilized fruitlets were lyophilized without any additional treatment.

For all samples, DNA extractions were performed from lyophilized samples using a modified version of the protocol described by Doyle & Doyle [26]. In brief, 20 mg of powdered (in liquid nitrogen) tissue were added to 800 µl of preheated (65°C) CTAB buffer (2% CTAB, 1% PVP, 100 mM EDTA pH 8, 100 mM Tris-HCl pH 8, 0,1% β- mercaptoethanol, 1.4 M NaCl) and incubated for 30 min at 65°C with periodic gentle swirling. The extraction mixture was cooled in ice and extracted twice with 800 µl of chloroform/iso-amyl alcohol (24∶1) and 500 µl of chloroform, respectively. DNA was precipitated with 2/3 volume of isopropanol (−20°C) and 1/10 volume of sodium acetate (3 M, pH 5.2) for 30 min at −20°C, washed with 70% cold ethanol (−20°C), dried and re-suspended in 100 µl of sterile distilled water. Total DNA from all olive samples was divided into two equal aliquots of 50 µl that were stored at −20°C without any additional treatment or after purification through chromatography columns as described [27].

### Evaluation of DNA quantity and quality

Purified environmental DNA samples were analyzed by electrophoresis in 1.5% agarose gel with GelRed nucleic acid Stain (Biotium, USA) in TBE buffer and visualized with UV light using Gel Doc (Bio Rad, USA). DNA concentration and quality was determined by measuring the absorbance at 260, 280 and 230 nm using a Nanodrop (Thermo Fisher Scientific Inc.). Furthermore, to confirm that DNA samples were of sufficient quality to be amplified by PCR, 1 µl of a representative number of DNA samples (purified and non-purified) was analyzed by real-time PCR using a specific hydrolysis probe method designed to detect *Phytophthora ramorum* [28]. Primers and probe for *P. ramorum* were selected because this species was not expected to be present in the olive phylloplane. Duplicate reaction mixtures contained 50 ng of *P. ramorum* DNA and were spiked with 1 µL of water (control) or either purified or non- purified DNA from olive samples. Reaction mixtures without *P. ramorum* DNA were utilized to confirm the absence of this species in all analyzed samples. PCR amplifications were performed as described by Schena and co-workers [28] using a StepOnePlus Real-Time PCR System (Applied Biosystems, USA) and data acquisition and analysis realized using the supplied software according to the manufacturer's instructions. The quantification cycle (Cq) value for each reaction was calculated automatically by the StepOnePlus software by determining the PCR cycle number at which the reporter fluorescence exceeded background [29]

### Design and validation of ITS genus-specific primers

Considerable effort was made to design primers that could be used under standardized conditions to amplify the ITS region from all *Colletotrichum* species avoiding where possible other related *Ascomycota*. A comprehensive range of validated sequences comprising the final part of the 18S gene, the complete ITS1-5.8S-ITS2 region and the initial part of the 28S gene was analyzed. Analyzed sequences comprised all those recently utilized to redefine the systematic of the genus *Colletotrichum* [1], [5]–[7], [30] and representative sequences from other relevant publications [8], [30]–[33]. The complete set of validated sequences was preliminarily analyzed with the ElimDupes software (http://hcv.lanl.gov/content/sequence/ELIMDUPES/elimdupes.html) to eliminate multiple identical sequences. Unique ITS sequences were aligned with representative sequences of phylogenetically related species [34] with the Multalin software [35] and manually examined to identify conserved regions within the genus *Colletotrichum*. A couple of degenerate primers (Coll1F (5'-AACCWGCGGAGGGATCATTA-3' and Coll3Rb 5'-TCCCTBCGRRTCCCRRTGCG-3') was designed in the final part of 18S gene and ITS2 region, respectively. This primer localization enabled the amplification of the almost complete ITS1-5.8S-ITS2 region. Primers were designed with the Primer 3 software [36] using a reference sequence of *C. godetiae* and degenerations were subsequently manually added according to the alignment of all *Colletotrichum* species.

Designed primers were validated using target DNA from 16 different *Colletotrichum* species and 13 related ascomycetes (Table 1). Amplifications were carried out in a total volume of 25 µl containing 1× PCR buffer, 0.1 mM dNTPs, 1.5 mM MgCl_2_, 1 unit *Taq* polymerase (Invitrogen, CA, USA), 0.5 µM for each primer and 1 µl of genomic DNA (50 ng). Optimized reaction conditions consisted of 3 min at 95°C followed by 35 cycles of 30 s at 95°C, 30 s at 55°C and 45 s at 72°C and by a final extension of 5 min at 72°C. All reactions were incubated in a Mastercycler Ep Gradient S. (Eppendorf, Germany). Amplicons were separated by electrophoresis in 1.5% agarose gel with GelRed nucleic acid Stain in TBE buffer and visualized with UV light as described above.

**Table 1:** List of species and isolates utilized to evaluate the specificity of *Colletotrichum*-genus-specific primers and corresponding positive (+) or negative (-) amplification results obtained in PCR reactions with pure culture DNA samples.

Fungal species	Isolate code	Host	Origin	Amplification
*Colletotrichum acutatum s.s.*	OLE	*Nerium oleander*	Sicily, Italy	+
*C. acutatum s.s.*	F29	*Olea europaea*	NSW, Australia	+
*C. aenigma*	C53	*Pyrus communis*	Apulia, Italy	+
*C. godetiae*	CBS 193,32	*O. europaea*	Greece	+
*C. godetiae*	OLF48	*O. europaea*	Apulia, Italy	+
*C. coccodes*	IMI1366011	*Lycopersicon esculentum*	New Zealand	+
*C. coccodes*	P. ALBENGA	*Capsicum annuum*	Piedmont, Italy	+
*C. destructivum*	BAS	*Ocimum basilicum*	Liguria, Italy	+
*C. fioriniae*	1491	*Vaccinium myrtillus*	Latium, Italy	+
*C. fioriniae*	OLPUGLIA	*Olea oleaster*	Smirne, Turkey	+
*C. gloeosporioides s.s*	S1/S2	*O. europaea*	Sicily, Italy	+
*C. gloeosporioides s.s.*	F108	*O. europaea*	Italy	+
*C. graminicola*	Gra1	*Zea mais*	USA	+
*C. kahawae* subsp. *ciggaro*	Isol. 53	*O. europaea*	Apulia, Italy	+
*C. karstii*	C18	*Citrus sp.*	Italy	+
*C. karstii*	OLF38	*O. europaea*	Italy	+
*C. musae*	MUSAE	*Musa x paradisiaca*	Sicily, Italy	+
*C. queenslandicum*	VMIN	*O. europaea*	Montenegro	+
*C. nymphaeae*	1036	*Cyclamen persicum*	Latium, Italy	+
*C. nymphaeae*	1567	*O. europaea*	Algarve, Portugal	+
*C. nymphaeae*	SPL100	*Fragaria × Ananassa*	Calabria, Italy	+
*C. theobromicola*	F27	*O. europaea*	NSW, Australia	+
*Colletotrichum* sp.	OL24	*O. europaea*	Italy	+
*Ampelomyces* sp.	F34	*O. europaea*	Queensland, Australia	-
*Aspergillus versicolor*	SEPL1815	n.d.	Calabria, Italy	-
*Cladosporium cladosporioides*	F109	*Ceratonia siliqua*	Sicily, Italy	-
*Cryphonectria parasitica*	CRYO	*Castanea sativa*	Calabria, Italy	-
*Fusarium* sp.	F98	*Capsicum* sp.	Piedmont, Italy	-
*Monilinia laxa*	MONILIA	*Prunus avium*	Apulia, Italy	-
*Phomopsis amygdali*	F134	*Amygdalus communis*	Apulia, Italy	-
*Phomopsis* sp.	F119	*Pinus* sp.	Abruzzo, Italy	-
*Pseudocercospora* sp.	F106	*Ceratonia siliqua*	Sicily, Italy	-
*Ramularia eucalypti*	F107	*Citrus sinensis*	Calabria, Italy	-
*Rosellinia necatrix*	F130	*Juglans regia*	Apulia, Italy	-
*Verticillium dahliae*	V39	*Olea europaea*	Apulia, Italy	-
*Trichoderma viride*	F117	*Pinus nigra*	Abruzzo, Italy	-

### Detection of *Colletotrichum* spp. in the olive carposphere and phyllosphere

Selected primers and DNA extraction procedures were utilized to analyze green and senescent leaves, floral residues, symptomatic and asymptomatic fruits, and litter leaves and mummies (Table 2). A total of 113 samples were collected during 2012 in three phenological phases (June, October and December) from eight plants (cv Ottobratica) located in two fields (T1 and T2) of a private farm (Taccone P.L., Rizziconi, Reggio Calabria, Italy) and in a field (A1) of a public agricultural experiment station (ARSAC, Azienda Regionale per lo Sviluppo Agricolo Calabrese, Gioia Tauro, RC) (Table 2). All fields were located on the Gioia Tauro Plain, Calabria, southern Italy and were representative of an olive-growing area of over 32,000 ha, where epidemic anthracnose outbreaks occur yearly [37]. Senescent leaves were characterized by general symptoms of decline (mainly discolorations and local necrosis) not directly ascribable to *Colletotrichum* infections. Symptoms on fruits collected from the canopy in December were considered more specific of *Colletotrichum* infections, even though it was not possible to completely exclude the role of other fungal pathogens such as *Fusarium* spp. For each olive tree 30–200 g of tissues (according to the kind of organs) were collected all around the selected plant at a height of approximately 2 m.

**Table 2:** Summary of results of field surveys conducted with different olive tissues collected in 3 phenological phases from 8 different plants located in three fields (T1, T2, A1).

Sampling date	Sample type	Field T1*			Field T2*		Field A1*		
		P1	P2	P3	P1	P2	P1	P2	P3
29.06.12	Green leaves	-	-	-	-	-	-	-	-
	Senescent leaves	-	-	-	-	-	Ca (0.7)	Ca (0.6)	-
							Cgo (0.2)	Cgo (0.3)	
							Cgl (0.1)	Cgl (0.1)	
	Floral residues	nd	Ca (1.0)	-	-	Cgo (0.6)	nd	nd	nd
						Ca (0.4)			
	Fertilized fruitlets	-	-	-	nd	-	nd	-	-
	Litter leaves	nd	-	-	-	-	-	-	-
									
17.10.12	Green leaves	-	-	-	-	-	-	-	-
	Senescent leaves	-	-	-	-	-	-	-	-
	Asymptomatic fruits	-		-	Ck (1.0)		-	-	-
	Litter leaves	-	-	-	-	-	-	-	-
									
12.12.12	Green leaves	-	Cgo (1.0)	Cgo (1.0)	-	Cgo (1.0)	-	Cgo (1.0)	-
	Senescent leaves	-	Ca (0.4)	Ca (0.5)	-	Cgo (0.9)	Ca (0.75)	-	-
			Cgo (0.4)	Cgo (0.3)		Ca (0.1)	Cgo (0.25)		
			Cgl (0.1)	Cgl (0.1)					
	Asymptomatic fruits	Cgo (0.6)	-	-	-	Cgo (1.0)	Cgo (0.6)	Cgo (1.0)	Ca (0.85)
		Ca (0.3)					Ca (0,4)		Casl (0.1)
		Cgl (0.1)							Cgsl (0.05)
	Symptomatic fruits	Cgo (0.6)	Cgo (0.8)	Ca (0.5)	Ca (0.7)	Cgo (1.0)	Cgo (1.0)	Cgo (0.9)	Ca (0.7)
		Cgl (0.3)	Ca (0.2)	Cgo (0.4)	Cgo (0.2)			Ca (0.1)	Cgo (0.1)
		Ca (0.1)		Casl (0.1)	Cbsl (0.1)				Casl (0.2)
	Litter leaves	Ca (0.6)	Ca (1.0)	Ca (0.6)	-	Cgo (1.0)	Ca (1.0)	-	-
		Cgo (0.4)		Cgo (0.4)					
	Litter mummies	Ca (0,6)	Cgo (1.0)	Ca (0.7)	-	Cgo (0.7)	Cgo (0.7)	Ca (0.8)	-
		Cgo (0,4)		Cgo (0.3)		Ca (0.3)	Cbsl (0.1)	Cgo (0.2)	
							Ca (0.2)		

*GPS coordinates: T1 (38°22'53.0"N, 15°56'27.5"E), T2 (38°22'15.1"N 15°55'38.3"E) and A1 (38°24'44.6"N, 15°56'23.1"E). (nd)  =  not analyzed samples; (-) analyzed samples that did not produce positive amplifications.

Detected phylotypes were associated with *Colletotrichum godetiae* (Cgo), *C. acutatum sensu stricto* (Ca), *C. gloeosporioides s.s.* (Cgl), *C. kahawae* (Ck), and non well-defined species of *C. acutatum* s.l (Casl), *C. gloeosporioides s.l.* (Cgsl) and *C. boninense s.l.* (Cbsl). Numbers in brackets represent the percentage of sequences associated with different phylotypes in each cloned PCR fragment.

### Amplification, cloning and sequencing of PCR fragments obtained from olive tissues

Extracted DNA samples obtained from each olive sample were amplified in triplicate with *Colletotrichum* spp. specific primers as described for pure fungal cultures. PCR products from each sample were merged, purified through chromatography columns (PCR Extract Mini Kit, 5Prime) and cloned into competent cells of *Escherichia coli* using the pGEM-T Easy Vector System (Promega, Milan, Italy) according to the manufacturer's protocol. For each cloned sample, at least 20 colonies were randomly picked and directly used in PCR reactions (colony PCR) with *Colletotrichum* specific primers as described above. Amplified fragments were analyzed by electrophoresis and sequenced with both forward and reverse primers by Macrogen Europe (Amsterdam, The Netherlands).

### Analyses of sequences and species identification

The ChromasPro v. 1.5 software (http://www.technelysium.com.au/) was used to evaluate reliability of sequences and to create consensus sequences. Unreliable sequences in which either forward or reverse sequences contained doubtful bases were resequenced. Prior to analysis, sequences of primers were removed.

Consensus sequences were aligned using the software MUSCLE [38] as implemented in MEGA5 [39] and edited manually for checking indels and single nucleotide polymorphisms. Genotypes, defined as the distinct and reproducible ITS sequences recovered in this study, were identified in MUSCLE and confirmed using the DnaSP ver. 5.10.01 software [40]. In order to reduce the risk of errors due to artifacts during PCR and/or plasmid replication, only genotypes represented by at least two sequences were considered for further analyses and deposited in GenBank with accession numbers KJ710682-KJ710696 (Table 3).

**Table 3:** List of *Colletotrichum* species and ITS genotypes identified in different olive tissues collected in three olive orchards on the Gioia Tauro plain (southern Italy).

**C. godetiae**									
Genotypes	SNPs						Samples*	Orchards	AN**
	43	125	133	307	338	369			
God1	G	A	T	A	C	C	27	T1, T2, A1	KJ710686
God2	.	.	.	.	.	A	8	T1, T2, A1	KJ710687
God3	A	.	.	.	.	.	2	T2, A1	KJ710688
God4	.	.	C	.	.	.	1	A1	KJ710689
God5	.	.	.	.	T	.	2	T1,	KJ710690
God6	.	.	.	G	.	A	1	A1	KJ710691
God7	.	G	.	.	.	.	2	T1	KJ710692
**C. acutatum s.s.**									
Genotypes	SNPs				Sample*		Orchards	AN**	
	318	427	429	430					
Acu1	T	-	A	A	20		T1, T2, A1	KJ710693	
Acu2	C	-	.	.	1		T1	KJ710695	
Acu3	.	A	T	T	1		T2	KJ710696	
**C. acutatum s.l.**									
Genotype	Samples*		Orchards		AN**				
Acusl	3		T1, A1		KJ710694				
**C. gloeosporioides s.s.**									
Genotype	Samples*		Orchards		AN**				
Glo	5		T1, A1		KJ710684				
**C. gloeosporioides s.l.**									
Genotype	Samples*		Orchards		AN**				
Glosl	1		A1		KJ710685				
**C. kahawae**									
Genotype	Samples*		Orchards		AN**				
Kah	1		T2		KJ710683				
**C. boninense s.l.**									
Genotype	Samples*		Orchards		AN**				
Bonsl	2		T2, A1		KJ710682				

*Number of samples in which each genotype was detected

**Accession numbers

**Figure 1:**
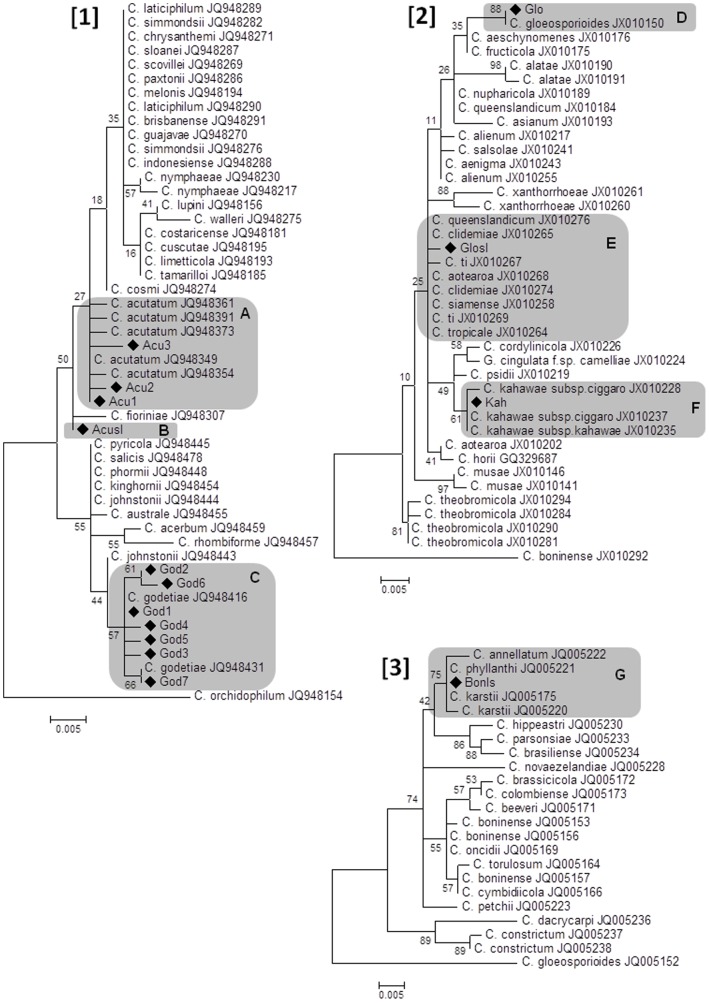
Phylogenetic trees built using unique sequences representative of all detected genotypes (♦) together with sequences of reference isolates of *Colletotrichum acutatum sensu lato* [1], *C. gloeosporioides s.l.* [2] and *C. boninense s.l.* [3]. Genotypes were identified as *C. godetiae* (A), *C. acutatum s.s.* (C), *C. gloeosporioides s.s.* (D) and *C. kahawae* (F). Three additional genotypes were associated with 2 (B), 6 (D) and 3 (F) species within *C. acutatum s.l.*, *C. gloeosporioides s.l* and *C. boninense s.l.*, respectively. Separate analyses were conducted for each species complex. Numbers on nodes represent the posterior probabilities for the maximum likelihood method.

The number of samples and the orchards (Cfr. Table 2) in which each genotype was detected is reported together with GenBank accession numbers for sequences. Genotypes were grouped according to their phylogenetic identification (Cfr. Fig. 1).

To identify species, single representative sequences of each detected genotype were phylogenetically analyzed along with validated barcode sequences of *C. acutatum s.l., C. gloeosporioides s.l.*, or *C. boninense s.l.* [1], [5]–[7]. Before analyses, the complete panel of *Colletotrichum* reference sequences were analyzed with the software ElimDupes (http://hcv.lanl.gov/content/sequence/ELIMDUPES/elimdupes.html) to delete multiple identical sequences. A few identical reference sequences were included in the panel because they were representative of different *Colletotrichum* species. When none of the above reference sequences was identical to genotypes identified in the present study, the existence of eventual more closely related sequences was evaluated by BLAST analyses.

Genotype and reference sequences were aligned using MUSCLE and introduced to MEGA for phylogenetic analysis with the Maximum Likelihood method using the Tamura-Nei model [39]. Analyses were performed with 1000 bootstrap replications.

To evaluate how different detected genotypes were correlated with each other within *C. acutatum s.l., C gloeosporioides s.l.* and *C. boninense s.l*., networks were generated with the statistical parsimony algorithm implemented in TCS ver. 1.21 [41]. In the networks different colors were utilized to associate genotypes with the different sampled periods and organs while the area of the pies was directly correlated with the frequency of each genotype i.e. the number of analyzed samples in which each genotype was detected.

## Results

### Validation of *Colletotrichum* spp. genus-specific primers

PCR reactions conducted with DNA samples from different species of *Colletotrichum* and related *Ascomycota* confirmed a high level of specificity for primers Coll1F-Coll3R that provided a bright positive amplification with target DNA from the complete set of *Colletotrichum* species but not from other fungal genera (Table 1). Compared to alternative markers developed in the present study, primers Coll1F-Coll3R enabled the amplification of the almost complete ITS1-5.8S-ITS2 region and provided the best compromise between specificity, efficient amplification and absence of primer-dimers (data not shown).

### DNA extractions from olive tissues

Extracted DNA proved to be suitable for PCR amplifications after the purification step with the chromatography columns. Prior to purification, some extracts were not completely clarified and caused a significant inhibition of real-time PCR reactions. The quantification cycle (Cq) of reaction mixtures spiked with 1 µl of non-purified DNA was generally retarded and, in some cases, reactions were completely inhibited (data not shown). Conversely, purified DNA samples did not cause any delay of the Cq against the positive control, confirming the absence of any inhibition of the Taq DNA polymerase activity. Reaction mixtures without exogenous DNA of *P. ramorum* did not produce any increase in fluorescence, thus confirming the absence of this pathogen in the analyzed samples. The concentration of nucleic acids in extracted samples ranged between 200 and 500 ng/µl while the 260/280A and 260/230A ratios ranged between 1.8 and 2.1 and between 1.3 and 2.0, respectively.

### Amplification results from olive tissues

A total of 113 olive samples were amplified with *Colletotrichum* spp. specific primers, 37 of which produced an evident positive amplification with PCR fragments of the expected size (Table 2). Faint PCR fragments were amplified from a few additional samples, even though the low quantity of amplicons did not enable the subsequent cloning and sequencing. Considering only evident positive amplifications, a single sample of asymptomatic fruits collected in October yielded a PCR fragment of the expected size (≅470 bp). A higher incidence of positive samples was revealed in June with two samples of senescent leaves and floral residues, respectively. Finally, the great majority of samples collected in December produced a positive amplification. In particular, PCR fragments of the expected size were obtained from all symptomatic fruits (Table 2).

### Analysis of sequences and species identification

**Figure 2:**
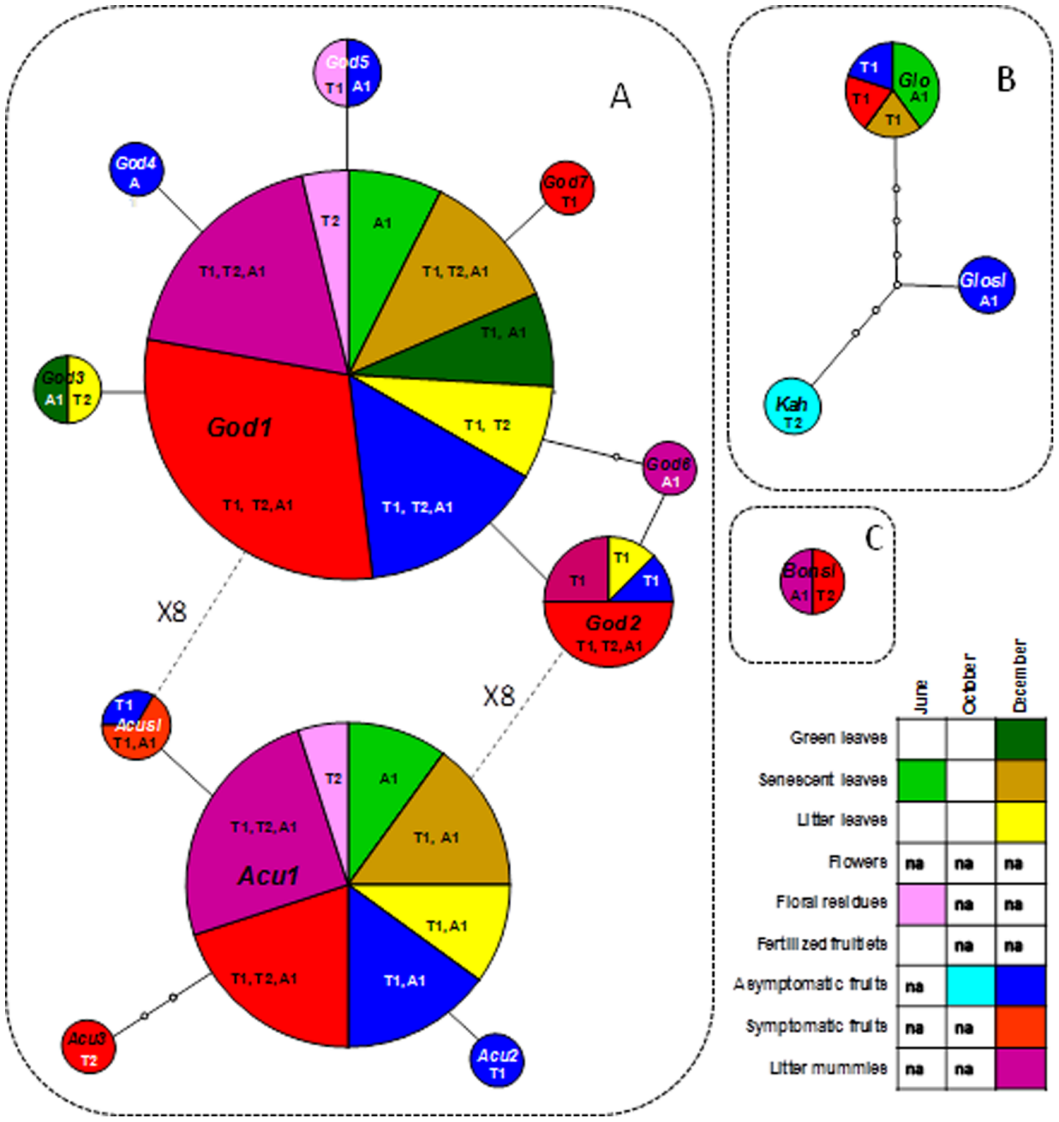
Genotype networks based on ITS sequences of *Colletotrichum acutatum sensu lato* (A), *C. gloeosporioides s.l.* (B) and *C. boninense s.l.* (C), detected in different olive tissues in 3 different phenological phases (June, October and December). According to the caption (bottom right of the figure) different colors were used to connect detected genotypes and analyzed olive samples. Empty white boxes in the caption indicate analyzed samples that did not produce any positive amplification, while white boxes containing “na” indicate non-analyzed samples. The letters “T1”, “T2” and “A1” inside the circles were used to indicate sampling fields where genotypes were detected (Cfr. Table 2). The size of each circle represents the relative frequency of genotypes in terms of number of samples in which they were detected. Genotypes were identified according to their phylogenetic collocation (Cfr. Fig. 1) and named using the initials of the corresponding species as follows: *C. godetiae* (Glo), *C. acutatum s.s.* (Acu), *C. gloeosporioides s.s.* (Glo), *C. kahawae* (Kah), *C. acutatum s.l.* (Acusl), *C. gloesporioides s.l.* (Glosl) and *C. boninense s.l.* (Bonsl).

The cloning of 37 positive PCR fragments from different olive tissues and the subsequent sequencing of 20 clones per sample yielded 740 reliable DNA sequences. The analysis of the complete panel of sequences enabled the identification of 15 different genotypes represented by at least two different sequences (Fig. 1; Fig. 2). All detected genotypes belonged to species of the genus *Colletotrichum.* Seven out of 15 genotypes (God1, God7, Acu1, Acu2, Glo, Kah, and Bonsl) were identical to reference sequences while the remaining 8 genotypes showed one or few polymorphic bases compared to currently available GenBank deposited sequences. However, genotypes perfectly matching reference sequences were much more diffused, accounting for the great majority of positive samples (Fig. 2).

The phylogenetic analysis of detected genotypes along with reference sequences permitted the identification of 6 *Colletotrichum* phylotypes clustering in three different species complexes (*C. acutatum s.l., C. gloeosporioides s.l.* and *C. boninense s.l.*). Genotypes clustering within *C. acutatum s.l.* were by far the most abundant (11), followed by genotypes of *C. gloeosporioides s.l*. (3) and a single genotype of *C. boninense.* Each phylotype was represented by a number of genotypes variable between 1 and 7 (Fig. 1; Fig. 2). Four phylotypes were identified at the level of species: *C. godetiae* (7 genotypes), *C. acutatum s.s.* (3 genotypes), *C. gloeosporioides s.s.* (1 genotype) and *C. kahawae* (1 genotype). For this latter genotype it was not possible to identify the subspecies since *C. kahawae* subsp. *ciggaro* and subsp. *kahawae* are characterized by identical ITS sequences [6]. Another phylotype, represented by a single genotype (Glosl) was associated with different species because its sequence was identical or very similar to that of 6 different recognized species (*C. aotearoa, C. queenslandicum, C. ti, C. tropicale, C. siamense, C. clidemiae*) within *C. gloeosporioides s.l.* (Fig. 1). Similarly, a phylotype represented by genotype "Bonsl" was associated with three different species (*C. karstii*, *C. phyllanthi*, *C. annellatum*) of the *C. boninense* species complex (Fig. 1). Finally, a genotype within *C. acutatum s.l.* (Acusl) was not identified at the level of species because closely related to both *C. acutatum s.s.* and *C. fioriniae* (Fig. 1).

### Diffusion of *Colletotrichum* species in olive samples

In the three investigated fields, *Colletotrichum* phylotypes were prevalently detected in olive samples collected in December (Table 2; Fig. 2). In this sampling period *Colletotrichum* spp. was detected in all samples of symptomatic fruit and in 6 out of 8 litter mummy samples. Furthermore, 5 out of 8 samples of litter leaves and 4 out of 8 samples of green and senescent leaves were found to be infected/contaminated by *Colletotrichum* species. A few additional positive samples comprised two samples of senescent leaves and two floral residues collected in June and a single sample of asymptomatic fruits collected in October (Table 2; Fig. 2).

Among detected phylotypes those associated with the species *C. godetiae* and *C. acutatum s.s.* were by far the most abundant and were detected in 36 out of the 37 positive samples (Table 1). In particular, these two phylotypes were detected in 32 and 26 samples, respectively, and in most cases (22 samples) both phylotypes were found in the same sample (Table 2). The *C. gloeosporioides s.s.* phylotype was detected in 6 samples including 4 samples of senescent leaves collected in June and December and two samples of symptomatic and asymptomatic fruits, respectively, collected in December (Table 2; Fig. 2). Furthermore, a phylotype of *C. acutatum* s.l. represented by a single genotype (Acusl) was detected in three samples of symptomatic or asymptomatic fruits collected in December. Finally, phylotypes represented by single genotypes of *C. boninense* s.l. (Bonsl), *C. gloeosporioides s.l.* (Glosl) and *C. kahawae* (Kah) were detected in one or two samples (Table 2; Fig. 2). Most phylotypes of *C. gloeosporioides s.l.* and *C. boninense s.l.* were detected in samples also infected/contaminated by phylotypes of *C. acutatum s.l.*. An exception was represented by a sample of asymptomatic fruits collected in October in which a single genotype of *C. boninense s.l.* (Bonsl) was detected.

## Discussion

In the present study, a metagenomic approach based on the use of genus specific primers was developed and utilized to characterize *Colletotrichum* spp. populations associated with the olive phyllosphere and carposphere in different phenological phases and in three different farms located on the Gioia Tauro Plain, southern Italy, where epidemic anthracnose outbreaks occur yearly [37]. Selected markers enabled the use of the ITS regions of the rDNA as a barcode gene for *in situ* species identifications. This region is widely accepted as a preferential fungal DNA barcode marker although it is not always discriminant as phylogenetically closely related species may have sequences identical or differing only by a few nucleotide positions [19], [42], [43]. In particular, metagenomic approaches are commonly based either on the ITS1 or ITS2 regions due to restrictions in sequence read lengths with second generation sequencing technologies, and their use in taxonomic analyses of fungal plant pathogens may be limited [44]. Conversely, primers developed in the present study enable the amplification of the almost entire ITS1-5.8S-ITS2 in order to provide more opportunities for species identification [45]. This aspect is particularly important for a genus like *Colletotrichum* in light of the high number of species that are sometimes characterized by identical or very similar ITS sequences [5]–[7].

*Colletotrichum* spp. specific primers were designed giving particular emphasis to their universality in order to amplify the target region from all currently recognized species of the genus *Colletotrichum* [1], [5]–[7], [30]–[33]. This strategy proved appropriate since positive amplifications were obtained in laboratory tests with all isolates of *Colletotrichum* but not from other fungal cultures. Furthermore, validation experiments conducted with olive samples yielded only sequences associated with the genus *Colletotrichum* spp. with a good level of discrimination given that 4 phylotypes (represented by 12 genotypes) were identified at the level of species (*C. godetiae, C. acutatum s.s.*, *C. gloeosporioides s.s.* and C. *kahawae*) and three additional phylotypes, represented by genotypes Acusl, Glosl and Bonsl, were associated with a restricted number of closely related species. The species *C. kahawae* comprises two subspecies, *kahawae* and *ciggaro*, that cannot be molecularly distinguished using ITS sequences [6]. However, the detected genotype is likely to belong to the subspecies *ciggaro* since it has been recently isolated from olives in Italy [8] while the subspecies *kahawae* is specifically ascribed to *Colletotrichum* isolates causing Coffee Berry Disease in Africa [47]. Similarly, the genotype Bonsl is likely to belong to the species *C. karstii* since it has been recently reported on olives in southern Italy, while the other two species clustering in the same ITS group (*C. phyllanthi* and *C. annellatum*) have never been associated with olive trees [8]. More uncertain is the identity of the genotype Glosl since it clustered with 7 different recognized species of *Colletotrichum* spp. of which two (*C. queenslandicum* and *C. siamense*) have been reported on olive in Montenegro and Australia, respectively [8]. The identity of a genotype of *C. acutatum s.l.* (Acusl) also remains since related to both *C. acutatum s.s.* and *C. fioriniae*. The use of a more variable marker as a barcode gene could enable a higher level of discrimination among species [5], [7]. However, the single copy nature of currently available marker genes is likely to provide lower levels of sensitivity compared to the multi-copy ITS regions. Furthermore, the lower number of available reference sequences in genetic databases may represent an issue in species identification [42].

A conventional cloning and Sanger sequencing approach was utilized in the present study to determine genotypes. Although this technique is much less powerful than second generation sequencing strategies and too expensive to analyze the whole fungal genetic diversity, it can still represent a valuable alternative when genus specific primers are utilized [23], [24]. An important advantage of the Sanger approach is the high reliability of sequences especially if, as in the present study, they are determined in both directions. Furthermore, it does not require advanced bioinformatics acquaintances for the analyses of data from second generation sequencing [46]. Nevertheless, the localization of primers developed in the present study (final parts of 18S gene and ITS2 region, respectively) significantly reduced the length of amplicons (≅470 bp) compared to common universal fungal primers and may facilitate their future uses in second generation sequencing approaches [20].

Quite a high number of *Colletotrichum* ITS1-5.8S-ITS2 genotypes (15) were detected within analyzed olive samples. In particular, the detection of different genotypes associated with *C. godetiae* (7) and *C. acutatum s.s*. (3) suggests the existence of a higher genetic variability within these species compared to previously available data. Indeed, low abundant genotypes are likely to remain undetected using traditional culturing approaches although it cannot be completely excluded that some of the detected genotypes were the result of sequence artifacts during amplifications [48]. Furthermore, one problem that is relatively unique to rDNA regions, including the ITS region, is the possibility of intra-genomic (within-individual) variability [49]. In this context it is important to highlight that procedural precautions were taken to increase the reliability of the data. Firstly, identified genotypes were represented by at least two sequences given that single sequences were excluded as a precautionary measure. Since the introduction of identical errors in two different sequences is an unlikely occurrence, it seems possible to exclude the existence of PCR artifacts introduced during the last two steps of the analyses (plasmid replication and colony PCR). Furthermore, the identification of the same genotypes in different samples (separate extraction and amplifications) support data accuracy for at least some of the detected genotypes (Fig. 2). It should also be considered that two of the genotypes detected in single samples (Kah and Glosl) were identical to GenBank deposited items and too far from all other detected genotypes to support their artifact's nature. Based on previous considerations, if on the one hand artifacts cannot be completely excluded, on the other hand it is also possible that the genetic diversity reported in the present study was still underestimated since some of the excluded single sequences could actually represent true rare genotypes.

Among detected phylotypes those associated with *C. godetiae* and *C. acutatum s.s.* were by far the most abundant with a slight prevalence of the first over the latter and, in most cases, both phylotypes were detected in the same sample. On the contrary, the *C. gloeosporioides s.s.* phylotype was detected in a limited number of samples and other phylotypes, associated with *C. karstii*, *C. kahawae* and to non well-defined species of *C. acutatum s.l.* and *C. gloeosporioides s.l.*, were rarely detected. These results were partially expected because both *C. acutatum s.l*. and *C. gloeosporioides s.l*. have been associated with olive anthracnose, but the first species complex is by far the predominant in most olive-growing regions [9], [12], [13], [50]. However, the detection of *C. acutatum s.s.* in the present study represent a novelty for Italy. According to previous available data the population of *Colletotrichum* associated with olive anthracnose in Italy was prevalently constituted by *C. godetiae* and to a reduced extent by *C. gloeosporioides* [2], [11]. *C. acutatum s.s.* was, however, known as a prevalent species in olive-growing areas in Australia and South Africa but until recently it had never been isolated from olive in Italy [5], [11], [51]. In the Mediterranean basin, *C. acutatum s.s.* was isolated from infected olives in Portugal, but it represented a conspicuous part of the population only in the Algarve region since in other part of Portugal *C. nymphaeae* and *C. godetiae* were the prevalent species [12], [50]. Many factors including climatic changes, imports of new pathogen strains from other geographic regions, an outbreak of pathogens already endemically present and the adaptation of pathogens to new hosts may have played a role in the emergence of *C. acutatum s.s.* in Italy.

Regarding *C. gloeosporioides s.s.* its low frequency in analyzed samples seem to confirm its secondary role in olive anthracnose, although recent investigations have revealed that some isolates of this fungus can be as virulent as *C. acutatum s.l.* species [8]. In the present study *C. gloeosporioides s.s.* was constantly detected with *C. godetiae* and/or *C. acutatum s.l*.. As a consequence, the evaluation of its role in the development of the disease in olive drupes is challenging. Finally, other *Colletotrichum* phylotypes detected in the present study should not constitute a serious threat for olive production taking into consideration their rare detection and their reported low virulence on inoculated olives. In particular *C. kahawae* subsp. *ciggaro* and *C. karstii* were characterized by a very low virulence and infected only overripe olives [8].

*Colletotrichum* species were largely detected in olive samples collected in December from all investigated organs (symptomatic and asymptomatic fruits, green and senescent leaves, and litter leaves and mummies) while a limited number of samples was found infected in previous sampling periods. Indeed, *Colletotrichum* spp. was only detected in two samples of both floral residues and senescent leaves in June while it was not detected in October since *C. karstii*, found in a single fruit sample, was considered of secondary importance [8]. The non-detection of relevant *Colletotrichum* species in October samples may be the result of a delayed epidemic outbreak of the disease due to hot and dry weather conditions in the early fall. However, the complete absence of the pathogen remains to some extent surprising since latent infections of developing fruits in the spring have been reported to favor the survival of the pathogen during the hot and dry summer and serve as an inoculum source for anthracnose epidemics on ripening fruit [14], [50]. Because the analyses of drupes conducted in present study was limited to the flesh (the stone was discarded before DNA extractions) it can be hypothesized that latent infections were not detected because the pathogen was mainly localized within the seeds [52]. Furthermore, latent infections of *Colletotrichum* species associated with olive anthracnose have been reported to occur with a very low incidence and as a consequence may not have been detected in this study just by chance or because their concentration in the hosts tissues was below the detection threshold of the technique [14]. In a previous study the pathogen was not isolated or was found with a very low incidence in naturally infected samples of fruits and leaves in June, July and August, although experiments were conducted in a region (Andalusia) severely affected by anthracnose [14]. Since the *Colletotrichum* species can produce a large number of conidia in conducive environmental conditions, the incidence of latent infections on drupes may be of secondary importance for the control of the disease. It has been observed that in conducive conditions only one infected drupe per tree can result in 100% affected fruits [14]. Furthermore, the absence of a correlation between spring primary infections and the incidence of the disease on fruits in late fall has been reported in both early and more recent studies [3], [53].

The non-detection of *Colletotrichum* species in October samples and its subsequent high incidence in all olive samples in December suggest that infections of olive fruits and leaves mainly occurred in late fall in coincidence with the ripening of fruits in conducive environmental conditions [54]. It is well documented that the susceptibility of the fruits to anthracnose increases with the progress of the maturation process and that the presence of fruits is also essential for leaf infections [13], [14]. Also, the presence of the pathogen in litter leaves and mummies is likely to be the result of earlier infections from the fall. According to previous reports, litter leaves and mummies should play a minor role in the production of inoculum compared to air mummies since they are rapidly degraded by secondary invaders and/or buried in the soil [37], [54]. However, considering that a small quantity of inoculum is enough to generate new epidemics, they could still play a role in the conservation of the pathogen especially in regions like southern Italy, where the no-till farming is a common agricultural practice.

Interestingly, *C. godetiae, C. acutatum s.s.* and *C. gloeosporioides s.s.* were also detected in 2 out of 4 analyzed samples of floral residues collected in June suggesting their saprophytic behavior in colonizing dead tissues. The colonization of floral residues may play a role in increasing pathogen populations and may also favor the establishment of latent infections on fruits as commonly occurs for typical necrotrophic fungi [55].

In conclusion, the metagenomic approach developed in the present study proved to be a valuable tool to study *Colletotrichum* spp. populations in environmental samples and to provide new information on disease cycles and aetiology of a complex disease such as olive anthracnose in southern Italy. The use of this method in specific well-defined sampling designs should greatly contribute to the definition of the epidemiology of olive anthracnose which is still controversial even if it has been studied since the first report of its epidemic outbreaks [56].
